# Impact of biospecimens handling on biomarker research in breast cancer

**DOI:** 10.1186/1471-2407-9-409

**Published:** 2009-11-24

**Authors:** Loris De Cecco, Valeria Musella, Silvia Veneroni, Vera Cappelletti, Italia Bongarzone, Maurizio Callari, Barbara Valeri, Marco A Pierotti, Maria Grazia Daidone

**Affiliations:** 1 Fondazione Istituto FIRC di Oncologia Molecolare (IFOM), Via Adamello16, 20139 Milano, Italy; 2 Department of Experimental Oncology, Fondazione IRCCS Istituto Nazionale dei Tumori, Via Venezian 1, 20133 Milano, Italy; 3 Department of Pathology, Fondazione IRCCS Istituto Nazionale dei Tumori, Via Venezian 1, 20133 Milano, Italy; 4 Scientific Directorate, Fondazione IRCCS Istituto Nazionale dei Tumori, Via Venezian 1, 20133 Milano, Italy

## Abstract

**Background:**

Gene expression profiling is moving from the research setting to the practical clinical use.

Gene signatures able to correctly identify high risk breast cancer patients as well as to predict response to treatment are currently under intense investigation. While technical issues dealing with RNA preparation, choice of array platforms, statistical analytical tools are taken into account, the tissue collection process is seldom considered.

The time elapsed between surgical tissue removal and freezing of samples for biological characterizations is rarely well defined and/or recorded even for recently stored samples, despite the publications of standard operating procedures for biological sample collection for tissue banks.

**Methods:**

Breast cancer samples from 11 patients were collected immediately after surgical removal and subdivided into aliquots. One was immediately frozen and the others were maintained at room temperature for respectively 2, 6 and 24 hrs. RNA was extracted and gene expression profile was determined using cDNA arrays. Phosphoprotein profiles were studied in parallel.

**Results:**

Delayed freezing affected the RNA quality only in 3 samples, which were not subjected to gene profiling. In the 8 breast cancer cases with apparently intact RNA also in sample aliquots frozen at delayed times, 461 genes were modulated simply as a function of freezing timing. Some of these genes were included in gene signatures biologically and clinically relevant for breast cancer. Delayed freezing also affected detection of phosphoproteins, whose pattern may be crucial for clinical decision on target-directed drugs.

**Conclusion:**

Time elapsed between surgery and freezing of samples appears to have a strong impact and should be considered as a mandatory variable to control for clinical implications of inadequate tissue handling.

## Background

Our understanding of the underlying molecular mechanisms in various human tumors has increased exponentially over the last decades due to the rapid development and application of technologies such as DNA microarrays and mass spectrometry-based proteomics. DNA microarray technology has markedly contributed to the comprehension of the complexity of pathways governing aggressiveness and treatment response of human neoplasias [1]. Further developments are expected as techniques are improving and allow the use of tiny amounts of tissue both frozen or even fixed and paraffin-embedded for comprehensive molecular analyses [2].

When comparing results from published microarray studies, differences in patient cohorts, treatment regimens, type of gene expression platform employed are usually taken into account, while procedures and timing related to the processes encompassed between surgical excision and freezing and/or fixation of the biological samples are poorly controlled. Such procedures, applied during sample handling may however significantly affect microarray data. In particular ischemia combined with room temperature storage due to the prolongation of the time elapsed between surgical removal and snap-freezing in liquid nitrogen is likely to alter gene expression patterns [3] as well as protein expression [4]. If this is the case the gene expression data may be modified by an external source of variability, and consequently represent the result of a complicated interplay between disease-associated gene and conditions of sample handling rather than a specific disease condition.

Despite the definition of strict operating procedures for collection of samples in tissue banks [5], pre-analytical procedures have been scarcely ever controlled during the daily routine. Such pre-analytical variation is probably not likely to impact results from comprehensive genome-wide profiling studies designed to select or discover genes linked to a particular pathological condition. In fact when employing whole genome arrays the pre-analytical noise may be compensated by the large number of investigated transcripts. However, in the case of validation of signatures or even more in the case of their use for clinical decision, according to FDA-approved commercially available tests as the OncoDx™ (Genomic Health, Redwood City, CA) and the MammaPrint^® ^(Agendia, Netherlands), it is very important to try to build gene signatures containing robust genes not affected by handling procedures and therefore to define which are the genes particularly prone to be modified by inadequate pre-analytical processing. Indeed the effect of inappropriate tissue handling is a critical issue not only for frozen samples, but also for fixed samples where the elapsed time between surgical removal and fixation adds technical variability to the possible alterations induced by fixation procedure.

Some studies have already addressed the issue in a number of human, rat and mouse tissues. Using real-time RT-PCR quantification in mouse liver specimens, Almeida et al [3] assessed the expression of six genes and showed their modulation under ischemic conditions both at two different temperatures mimicking surgical ischemic conditions and at room temperature waiting time prior to pathological examination. Similarly using cDNA microarrays three separate groups, Huang [6], Blackhall [7] and Dash [8], analyzed respectively specimens from a human colon normal mucosa sample, a couple of lung tumors and four prostate samples. All these studies disclosed differential gene expression patterns related to delays in tissue processing. Miyatake et al [9] drew the same conclusion investigating the effects of ischemia in different tissues of rat (lung, liver, kidney and spleen). In addition, the authors demonstrated a tissue-dependent transcriptional response against warm ischemia stressing this way the importance of homogeneous surgical procedures for result comparison.

Breast tumors have been extensively profiled by gene expression analysis and encouraging data are available both in the area of prognosis and treatment response prediction [10]. In order to gain awareness of the gene expression artifacts linked to improper tissue handling also for this particular type of neoplasia, we have investigated gene expression profiles in a set of primary breast tumors, grossly subdivided into 4 aliquots and kept at room temperature for 0, 2, 6 and 24 hours after resection before snap-freezing in liquid nitrogen. In addition, we concomitantly explored the time-related preservation of tyrosine phosphorylated proteins, considered to be crucial in cancer development and directly implicated on target-directed therapeutics. Our results show that elapsed time between surgery and freezing has a strong effect on functional genomic analysis and may affect the expression of genes relevant for predictive signatures as well as the phosphorylation status of molecular targets. Therefore pre-analytical conditions should be taken into account as a separate variable both when designing and interpreting comprehensive molecular analyses on archival specimens.

## Methods

### Case material

Eleven histologically confirmed primary breast tumors were obtained from the Tissue Bank of the Fondazione IRCCS Istituto Nazionale Tumori between October 2006 and March 2007. For each sample a written informed consent signed by the patient was available authorizing the use of leftover material for research purposes. The study was approved by Independent Ethics Committee and Institutional Review Board. Samples used in the study were selected based on the size, carefully avoiding necrotic areas, fat and normal tissue. An adjacent section was stained and used for defining the percentage of tumor cells. Only specimens with more than 70% of tumor cells were included in the study. Each tissue sample was divided into 4 aliquots. One was immediately frozen while the remaining three were frozen after 2, 6 and 24 hours at room temperature.

### RNA isolation and expression profiling

Tissue was pulverized using a Mikrodismembrator (Braun Biotech International, Germany). Total RNA was extracted with the Trizol reagent (Invitrogen, Carlsbad, CA) according to manufacture instructions and an additional DNase digestion was performed using the RNeasy kit (Qiagen, Valencia, CA). After each extraction a small fraction of RNA was used for quality and yield assessment. RNA total concentration and purity were determined by UV spectrometry. Total RNA electrophoretic profile was analysed by the Agilent RNA 6000 NanoLabChip kit on the Agilent 2100 Bioanalyzer (Agilent Technologies, Palo Alto, CA) using the software provided by the manufacturer for determination of RIN (RNA integrity number).

Probe labelling and hybridization were performed as previously described [11]. The samples and a reference RNA (Universal Human Reference RNA, Stratagene, La Jolla, CA) were labelled directly with Cy3-dCTP (reference RNA) or Cy5-dCTP (sample RNA) (Amersham Biosciences, Little Chalfont, UK) and indirectly with 3DNA Submicro Expression Array Detection kit (Genisphere, Montvala, NJ). Hybridization was carried out in a hybridization station (Genomic Solutions, Ann Arbour, MI), slides were scanned using the GenePix 4000B microarray scanner and quantified using GenePix Pro 5.0.1.24 (Axon Instruments/Molecular Devices). The RNAs were hybridized on two different cDNA microarrays containing a total of 17172 unique clones selected from the Human sequence-verified I.M.A.G.E. clone collection (Research Genetics/Invitrogen, Carlsbad, CA) and spotted in triplicate. Raw gene expression data have been deposited at the European Bioinformatics Institute (EBI) ArrayExpress and are accessible through accession no. E-MEXP-2035.

### Quantitative real-time PCR

Total RNA isolated for the microarray analysis was used to verify the quantity of specific messengers by real-time PCR for 4 biologically relevant differentially expressed genes (i.e., FGFR4, ESR1, ERBB2, FBLN2). RNA was reverse transcribed using the High-Capacity cDNA Archive Kit (Applied Biosystems, Foster City, CA). Samples were amplified in multiplex PCR reactions using one of the assays of interest labelled with FAM. PPIA was used as housekeeping gene. Reactions were performed in a final reaction volume of 20 μl containing cDNA template, 10 μl 2× TaqMan Gene Expression Assay (Applied Biosystems, Foster City, CA) and thermal cycling was performed on an ABI PRISM 7700 Sequencer Detector (Applied Biosystems, Foster City, CA).

### Protein Profile Analysis

Following tissue pulverization, tumor samples were processed as described in [12]. Briefly, samples were lysed in an ice-cold buffer containing 50 mM HEPES (pH 7.6), 150 mM NaCl, 10% glycerol, 1% Triton X-100, 1.5 mM MgCl_2_, 1 mM EGTA, 10 mM Na_4_P_2_O_7_, 100 mM NaF, protease inhibitors and in presence or absence of 1 mM sodium orthovanadate. After 30 min incubation with gentle rocking at 4°C, lysates were cleared by centrifugation (10 min at 13,000 rpm). Supernatants were collected and, after protein concentration determination, tested for the presence of specific proteins by Western blotting. For Western blotting, cell lysates were resolved by 4-12% SDS-PAGE (precast gel NuPAGE, Invitrogen). Proteins were transferred to nitrocellulose membranes (Hybond-C Super, Amersham Bioscience, Little Chalfont, UK), checked for equal sample loading by Red Ponceau S staining, and probed with the appropriate antibodies. Immunoreactive bands were visualized using horseradish peroxidase-linked secondary antiserum (Amersham Biosciences, Little Chalfont, UK) and detected using an enhanced ECL system (Amersham Biosciences, Little Chalfont, UK). For Western blotting, the following antibodies were used: anti-p-Tyr 4G10 (Upstate Biotechnology, Millipore Corporation, Billerica, MA); p-Neu (Tyr 1248)-R (Santa Cruz, Biothecnology, Santa Cruz, CA) for ERBB2 and anti-FAK (Transduction Laboratories, BD, Franklin Lakes, NJ).

### Data analysis and Statistics

Raw microarray images and quantifications were stored and processed in Bio Array Software Environment (BASE, Lund Sweden) [13]. Poor signal quality of background-corrected Cy3 and Cy5 intensities were flagged and a lowess normalization [14] was applied to each slide. Replicated spots were averaged and their log (base2) expression ratios (tumor/reference) were downloaded from BASE and imported into BRB-ArrayTools version 3.4.0. (Bethesda, MD) developed by Richard Simon and Amy Peng Lam [15]. ANOVA analysis was performed following their instructions. All I.M.A.G.E clones annotations were updated with the latest release of NCBI Unigene (Build No. 194) using SOURCE[16]. In order to find the overlap between our list of genes and genes included in public available signatures, we employed the Merge function of the web-application MatchMiner [17]. Hierarchical clustering was performed using centered correlation and average linkage method.

For gene function analyses, a score obtained calculating the -Log(p-value) of the Fisher Exact Test indicates the probability that a function is obtained by chance; scores equal or greater than 3 give a 99.9% confidence level of not being generated by chance.

The genes identified by microarray analyses were challenged for gene function. After having imported their accession numbers into the Ingenuity Pathway Analysis (IPA) software (Ingenuity Systems, Mountain View, CA), they were categorized according to the gene functions present in the database.

Data analysis for real-time PCR was done using the Sequence Detector version 1.9 software. Relative log-expression of the genes (-ΔC_t_) was obtained subtracting the number of cycle threshold observed for the 18S gene from that observed for the gene of interest. After being exported further data analysis was performed in a BRB_ArrayToolv3.4.0.

The association between gene expression evaluated by cDNA microarry and real-time PCR was evaluated by linear regression analysis.

## Results and Discussion

### Gene expression analysis

The number of clinical reports dealing with gene expression profiling in human breast cancer has enormously increased, but little research has been performed to evaluate the variability due to sampling of surgical specimens, and no information has been generally provided about the time elapsed between surgical excision and tissue freezing. We addressed the problem by investigating on 11 individual breast tumors, whose clinico-pathological characteristics are shown in Table 1, the gene expression profile of 4 serial samples frozen at different time intervals from surgical resection.

**Table 1:** Clinico-pathological characteristics of samples

ID	ER status*	PR status^§^	ERBB2 status^	Age	Size (cm)	N status
Pb1	+	+	2+	69	6	+
Pb2	+	+	2+	85	7	-
Pb3	+	+	2+	30	2.5	+
Pb4	+	+	2+	46	1.1	-
Pb5	+	+	3+	58	2.0	+
Pb6	+	+	-	55	2.0	+
Pb7	+	+	2+	57	3.0	+
Pb8	-	-	-	40	4.0	-
Pb9	+	+	1+	43	6.0	+
Pb10	+	+	-	54	2.2	-
Pb11	+	-	-	80	5.5	-

*determined by ER-ICA

^§^determined by PgR-ICA

^determined by Hercept test (DAKO)

**Figure 1:**
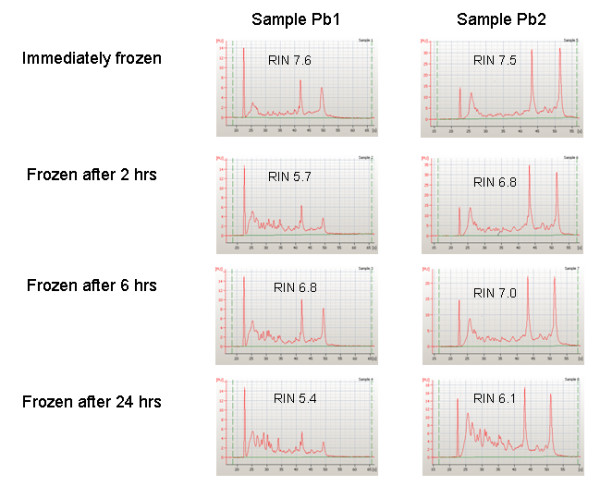
**RNA quality check**. RNA electropherograms obtained by running 250 ng of total RNA from aliquots of two representative samples on a 2100 Bioanalyzer with the RNA 6000 Nano LabChip. Elapsed time between surgical removal and freezing in liquid nitrogen is reported along with the RIN value. Each electropherogram reports fluorescence units as a function of running seconds.

**Figure 2:**
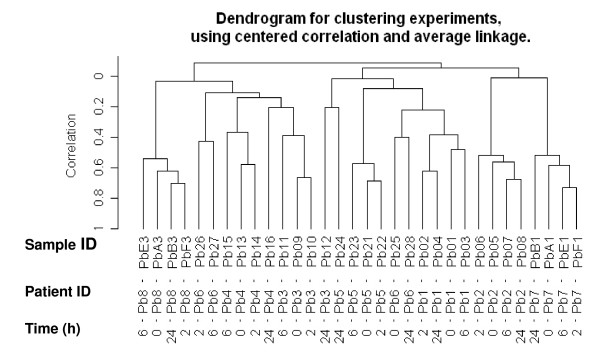
**Hierarchical clustering of all samples at different freezing times**. Hierarchical clustering of the gene expression matrix containing no missing values of 4 different aliquots derived from 8 individual samples. The dendrogram for clustering was obtained using centered correlation and average linkage. Patient ID and time elapsed between surgical removal and freezing are reported for each sample.

The evaluation of RNA showed a time dependent decrease of 28S and 18S ribosomal bands and a trend towards lower RIN numbers for samples stored for longer times at room temperature. Two examples are shown in Fig. 1, one with limited and one with more extended RNA degradation. Overall, for 8 breast cancers the serial samples showed valuable results, i.e., RNA not degraded (RIN>5) after prolonged exposure of samples to room temperature, and these cases were submitted to gene expression analysis [18,19]. Unsupervised hierarchical clustering analysis showed that samples belonging to the same patient tended preferentially to cluster together (Fig. 2), highlighting the patient-specific gene expression features despite different tissue freezing times. In fact only in 3 of the 8 samples (Pb6, Pb3 and Pb5) aliquots frozen at different times clustered separately.

Notwithstanding the relatively limited RNA degradation, a time-dependent modulation of genes was observed in the different serial aliquots, likely expected as a cellular reaction to stress conditions following the surgical resection of blood vessels and the suboptimal pre-analytical conditions. The gene expression profile of these serial specimens was performed using cDNA arrays containing 17,172 unique clones and analyzed by the ANOVA model. Using such approaches, genes whose modulation varied over the time independently of the intrinsic patient-specific differences were disclosed. A separate ANOVA model at significant threshold of p < 0.01 was fitted for each gene to relate the log-ratios expression to the factors under investigation (i.e. the time points and the different patients, the latter considered as blocking factor). Our results pointed out a significant variation as a function of time. Considering the number of genes modulated at each single time point compared to the sample immediately frozen as baseline, we noticed an increase in the number of modulated genes especially after 24 hours: 121 genes (0.76% of genes present in our chip) were modulated after 2 hours (66/121 were up-regulated); 157 genes (0.98%) were modulated after 6 hours (110/157 were up-regulated), 657 genes (4.1%) were modulated after 24 hours (287/657 were up-regulated) following tissue resection. Moreover, taking into account the overall time course of gene expression, we found 461 genes regulated at the significant threshold of p < 0.01, representing 2.88% of genes present in our chip. Around 1.03% of 461 genes were expected to be false positives according to the multiple testing correction method of Benjamini-Hochberg [20].

This result is in keeping with published data [3,4,6-9] in which a certain degree of gene expression variation as a function of collection procedures is reported. However, the modulation we noticed in breast cancer specimens was not as remarkable as the one reported by in Spruessel et al [4], where 20% of all detectable genes are modulated after 30 minutes of colon resection. In our study the majority of the genes (about two-thirds) were down-regulated, and up-regulated genes represented less than 1% of all the genes in our chip. Similarly Dash et al reported less than 0.6% of over-expressed genes in their study on radical prostatectomy specimens [8].

To better assess the impact of sample handling on the expression of critical genes, some of the modulated genes with an established biological role in breast cancer were subjected to a technical validation by qPCR. The statistically significant correlations between expression evaluated by cDNA microarray and Real Time PCR for FGFR4 (r = 0.57, p = 0.0008), FBLN2 (r = 0.595, p = 0.0004), ESR1 (r = 0.87, p = 0.0000008), ERBB2 (r = 0.754, p = 0.000002) clearly supports the fact that such a modulation is not imputable to technical artifacts, but represents a real biological phenomenon.

### Effect of sample collection procedure on gene pathways

**Figure 3:**
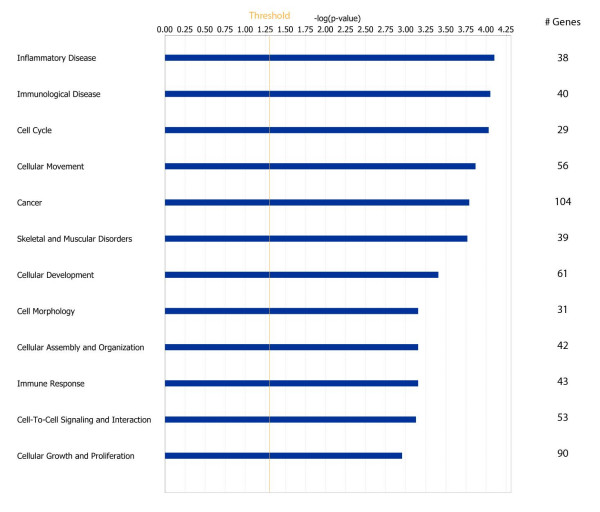
**Significantly enriched biological categories**. Chart of the most significantly enriched biological categories as identified with Ingenuity. For each category -log(p value) and number of genes are reported.

The 461 genes modulated as a function of delayed freezing of samples were analyzed using the IPA tool to understand if they were representative of specific biological categories. We focused our attention on the twelve most significant gene functions (Fig 3) that contain 69.6% of all genes which were present in the IPA database. Inflammatory, immunological disease, and cell cycle were the most significantly enriched gene classes. Globally various genes crucial for breast cancer were included. Among them, PIK3CD (p110δ) which is a catalytic subunit member of the class IA PI3K heterodimer, a signal transduction enzyme that regulates a broad spectrum of cellular functions as cell growth, proliferation and ultimately survival [21]. Inhibition of PIK3CD activity reduces VEGF activity leading to lack of vascular permeability [22]. As survival pathways play a crucial role both in prognostic outcome of breast cancer as well as in affecting treatment sensitivity, an artifact induction of such genes could be dramatically misleading. Similarly, among the genes down-regulated, STAT1 transcription was found repressed because of hypoxia mediated by HIF-1 and STRA13 [23]. CD44 was also among the down-regulated genes. This gene encodes a cell surface receptor for hyaluronate and is a direct target of miR373/miR520c, two recently identified metastasis-promoting microRNAs in breast cancer [24]. CD44 expression is associated with increased survival in node negative breast cancer [25] and decreased metastatic invasion [26].

**Figure 4:**
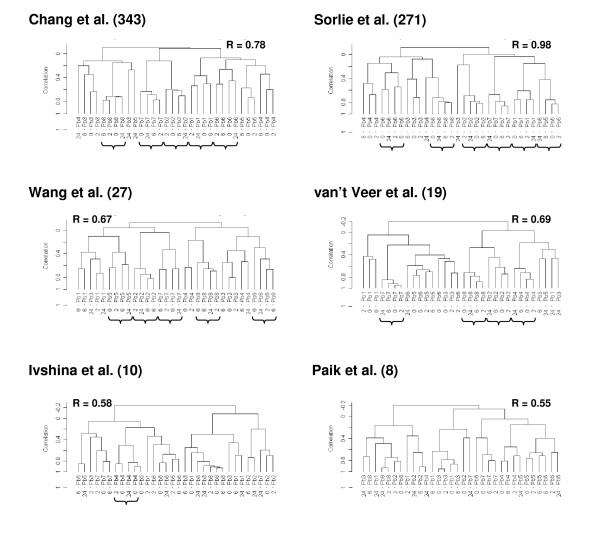
**Hierarchical clustering of all samples using literature signatures**. The number of genes common between our data set and each specific signature is reported in brackets. The dendrogram for clustering was obtained using centered correlation and average linkage. Patient ID and time elapsed between surgical removal and freezing are reported for each sample. The stability of our clusters (at a correlation level r>0.2) was investigated exploiting a BRB algorithm which performs 100 permutations of data and calculates an R index ranging between 0 and 1 where 0 indicates non reproducibility and 1 complete reproducibility. R index values are reported for each single clustering result.

Also genes reported to be associated with response to antiestrogen were found to be down-regulated by delayed tissue processing. Among them FBLN2, a gene reported to be up-regulated in elderly breast cancer patients who are not responsive to pre-operative treatment with toremifene [11] and FGFR4 whose up-regulation predicts poor response to tamoxifen [27]. Results are reported in Fig 4 panel A.

### Comparison with published breast cancer microarray studies

The use of gene signatures for predicting prognosis and response to treatment is quickly moving from a research area to the clinical practice. The reliability of such tools as well as they performance are strictly related to accurate gene expression determination which could be affected by sample handling and storage. To better understand to which extent commonly used signatures in breast cancer could be affected by artifacts linked to sample handling we verified how many of our 461 genes were included in such gene lists [2,28-33].

Results are reported in Table 2. No common genes were found between our 461 genes modulated by tissue handling conditions, two clinically relevant lists of genes associated to grading [29,31] and with the 70-gene classifier [32]. Conversely 28 genes were included in the intrinsic gene list [28], 24 in the wound healing gene list [27] and 17 genes were included in the new 1300-intrinsic gene list [33] and 2 in the list by Wang et al [34]. Genes included in such signatures were involved in lipid metabolism, transport, and cell proliferation.

**Table 2:** Genes common with main breast cancer genes signatures

28 genes common with the intrinsec genes list (Sorlie et al.)		
**P value**	**Gene symbol**	**Descriptor**

0.0003	FGFR4	Fibroblast growth factor receptor 4
0.0007	SELENBP1	Selenium binding protein 1
0.0010	POSTN	Periostin, osteoblast specific factor
0.0010	CALU	Calumenin
0.0012	SERPINH1	Serine (or cysteine) proteinase inhibitor, clade H (heat shock protein 47), member 1, (collagen binding protein 1)
0.0012	KDELR3	DEAD (Asp-Glu-Ala-Asp) box polypeptide 17
0.0013	PTMS	Parathymosin
0.0013	HIST2H2BE	Histone 2, H2be
0.0015	TUSC3	Tumor suppressor candidate 3
0.0016	ZNF516	Zinc finger protein 516
0.0018	BMI1	Polycomb group ring finger 4
0.0024	ACAD11	Putative acyl-CoA dehydrogenase
0.0026	INPP4B	Inositol polyphosphate-4-phosphatase, type II, 105 kDa
0.0033	ESR1	Estrogen receptor 1
0.0037	ACAA2	Myosin VB
0.005	CYB5A	Cytochrome b-5
0.005	CLIP4	CAP-GLY domain containing linker protein family, member 4
0.0051	AYTL1	Hypothetical protein FLJ20481
0.0056	RALGPS1	Ral GEF with PH domain and SH3 binding motif 1
0.0062	PSMB10	Proteasome (prosome, macropain) subunit, beta type, 10
0.0067	LRBA	LPS-responsive vesicle trafficking, beach and anchor containing
0.0070	UBE1	Ubiquitin-activating enzyme E1 (A1S9T and BN75 temperature sensitivity complementing)
0.0084	FBP1	Fructose-1,6-bisphosphatase 1
0.0087	COL6A3	Collagen, type VI, alpha 3
0.0087	TM9SF2	Transmembrane 9 superfamily member 2
0.0090	TAP1	Transporter 1, ATP-binding cassette, sub-family B (MDR/TAP)
0.0097	DIP2B	KIAA1463 protein
0.0100	TncRNA	Trophoblast-derived noncoding RNA

**24 genes common with wound healing signature (Chang et al)**		

0.0038	EIF4G1	Eukaryotic translation initiation factor 4 gamma, 1
0.0086	PRIM2	primase, polypeptide 2A, 58 kDa
0.0021	POR	P450 (cytochrome) oxidoreductase
0.0002	FANCG	Fanconi anemia, complementation group G
0.0097	EXOSC7	Exosome component 7
0.0006	FDPS	Farnesyl diphosphate synthase (farnesyl pyrophosphate synthetase, dimethylallyltranstransferase, geranyltranstransferase)
0.0020	C5orf4	Chromosome 5 open reading frame 4
0.0055	CDCA4	Cell division cycle associated 4
0.0099	H2AFV	H2A histone family, member V
0.0061	FABP3	Fatty acid binding protein 3, muscle and heart (mammary-derived growth inhibitor)
0.0027	CKLF	Chemokine-like factor
0.0014	MGC4308	Hypothetical protein MGC4308
0.0009	FADS1	Fatty acid desaturase 1
0.0061	SFTPB	Surfactant, pulmonary-associated protein B
0.0055	PLA2R1	Phospholipase A2 receptor 1, 180 kDa
0.0007	SELENBP1	Selenium binding protein 1
0.006	FARSB	Phenylalanine-tRNA synthetase-like, beta subunit
0.0054	MEF2D	MADS box transcription enhancer factor 2, polypeptide D (myocyte enhancer factor 2D)
0.0029	NUTF2	Nuclear transport factor 2
0.0098	ID3	Inhibitor of DNA binding 3, dominant negative helix-loop-helix protein
0.0087	ID2	Inhibitor of DNA binding 2, dominant negative helix-loop-helix protein
0.0069	PRG2	Proteoglycan 2, bone marrow
0.0001	H2AFX	H2A histone family, member X
0.0047	WSB1	WD repeat and SOCS box-containing 1

**17 genes common with new intrinsic gene list (Zhiyan et al.)**		

0.0033	TRIM33	Tripartite motif-containing 33
0.0003	RAB5C	RAB5C, member RAS oncogene family
0.0086	PRIM2	primase, polypeptide 2A, 58 kDa
0.0071	RAB21	RAB21, member RAS oncogene family
0.0029	SSTK	Serine/threonine protein kinase SSTK
0.0001	ST6GAL1	ST6 beta-galactosamide alpha-2,6-sialyltranferase 1
0.0052	IRF4	Interferon regulatory factor 4
0.0067	LRBA	LPS-responsive vesicle trafficking, beach and anchor containing
0.0036	IL13RA1	Interleukin 13 receptor, alpha 1
0.0033	ESR1	Estrogen receptor 1
0.0017	MFAP2	Microfibrillar-associated protein 2
0.0031	STAT1	Signal transducer and activator of transcription 1, 91 kDa
0.0013	PPFIA1	Protein tyrosine phosphatase, receptor type, f polypeptide (PTPRF), interacting protein (liprin), alpha 1
0.0061	MYO1C	Myosin IC
0.0082	PPFIA4	Protein tyrosine phosphatase, receptor type, f polypeptide (PTPRF), interacting protein (liprin), alpha 4
0.0060	RAB31	RAB31, member RAS oncogene family
0.0026	INPP4B	Inositol polyphosphate-4-phosphatase, type II, 105 kDa

**2 genes common with Recurrence Score genes (Paik et al)**		

0.0033	ESR1	Estrogen receptor 1
0.0021	MMP11	Matrix metalloproteinase 11 (stromelysin 3)

We then assumed that the extent of the impact of tissue handling on the prognostic/predictive reliability of a given gene signature, could be indirectly evaluated looking at its capability to maintain the clustering of individual samples collected at different times.

Results are reported in Fig. 4 for various gene signatures. After clustering the sample aliquots with the genes derived from the signature of Chang et al (343 genes in common with our data set) aliquots from 5 of 8 samples clustered together and similar data were obtained using the signature of Sorlie et al (271 genes in common), where in 6 out of 8 samples aliquots collected at different times clustered together. As expected, clustering of samples using signatures with a low number of genes as that of Paik et al [35], or with a small number of genes after searching for common genes within our data set, as that of Ivshina et al [31], were instead affected by inappropriate tissue handling as tissue aliquots obtained from the same patient did not cluster together. This underlines the fact that tissue mishandling has a limited effect on signatures with high gene numbers as already suggested in the *Background *section.

Interestingly, using the signatures with an intermediate number of genes (respectively 27 and 19 common genes) as in the case of the signature by Wang et al [34] and by van't Veer [32], we still observed that in most samples (5 out of 8 and 4 out of 8, respectively) aliquots from the same patient obtained at different times, did in fact cluster together. In these dendrograms the relatively low number of genes was probably partially compensated by the lack of overlapping (only 2 overlapping genes with the genes by Wang et al and none with genes by van't Veer) between such genes and the 461 genes that we identified as genes modulated by temperature. This may be an indirect proof of the fact that our 461 genes may be considered as reliable indicators of tissue mishandling

**Figure 5:**
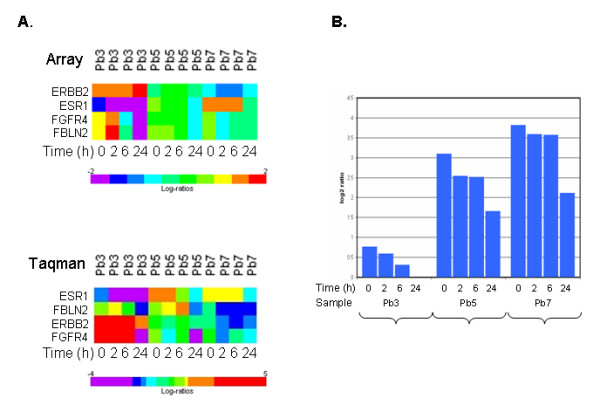
**Expression of selected genes as a function of time**. Panel A. Time course of ESR1, FBLN2, FGFR4 and ERBB2 expression for the three specimens (Pb3, Pb5 and Pb7). ESR1, FBLN2, FGFR4 are significantly down-modulated both in microarray and qPCR data. Panel B. Time course of ESRI microarray expression data for three samples with different basal ESR1 levels.

We also considered the expression of single genes implicated in breast cancer and currently used as predictive markers in the clinical management (ESR1, ERBB2, FBLN2, FGFR4, PGR). As reported in Fig 5 independently of ER base levels, ESR1 was consistently down-regulated through the four considered time points with the maximal down-regulation after 24 hours at room temperature. Fig 5 panel B reports the ESR1 microarray intensity expression for three samples where the effect is more evident (Pb3, Pb5 and Pb7). A similar down-regulation was observed for FBLN2 and FGFR4. On the contrary, no significant modulation was found for ERBB2.

### Impact of sample collection procedures on protein profile

**Figure 6:**
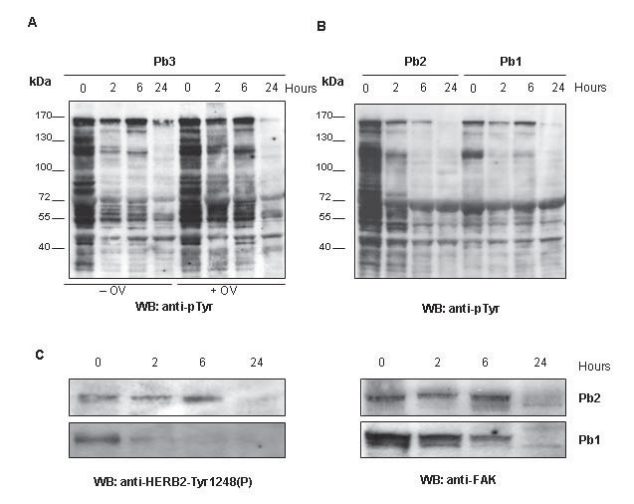
**Phosphotyrosine profiles as a function of freezing time**. Time course analysis (0, 2, 6 and 24 hours) of tyrosine phosphoproteins of three breast cancers. A. Anti-p-Tyr immunoblot on equal amounts of extracts from sample 1 lysed in absence or presence of Orthovanadate (OV); B. Anti-p-Tyr immunoblot on equal amounts of extracts from sample 2 and 3 lysed in presence of Orthovanadate; C. Anti-HERB2-Tyr1248(P) and anti-FAK immunoblots of equal amounts of extracts from sample 2 and 3.

In order to set up conditions to study signaling pathways in post resection specimens, we also explored the effects of pre-analytical procedures on protein status, examining tyrosine phosphorylation or expression in Western blotting experiments and time course conditions. We firstly explored the tyrosine phosphoprotein profiles testing the reactivity to antiphosphotyrosine antibody on total extracts of 2 serial samples from a breast cancer specimen (Pb2, Pb3) frozen at different times from surgery and obtained in absence or in presence of orthovanadate, a phosphatase inhibitor. Results are shown for Pb3 taken as a representative sample in Fig 6A. With the addition of orthovanadate in lysis buffer, the loss of phosphotyrosine proteins due to delayed freezing was clearly prevented up to 6 hours. However, after 24 hours at room temperature no major differences were observed between the pattern of phosphoproteins in the presence and in the absence of orthovanadate. These results provide the information that phosphatase inhibition is not sufficient to stabilize protein phosphorylation at room temperature. We then performed time course experiments on other two serial samples from breast cancers (Pb1 and Pb2) lysed in presence of orthovanadate (Fig 6B). Antiphosphotyrosine hybridization results highlighted the progressive loss of phosphoproteins during time course with individual variability probably depending on which specific phosphoprotein pathways become activated or suppressed *ex vivo*.

Detection of phosphoepitopes is often very important for understanding whether a specific pathway is active, thus inadequate pre-analytical procedure may dramatically modify biological interpretation of data obtained on clinical samples. Therefore sample Pb1 and Pb2, for which ERBB2 immuhistochemistry positivity was detected (Table 1) and gene expression analysis showed no significant change as a function of the time delay in freezing, were tested in Western blotting for the level of Tyr 1248 phosphorylation of ERBB2 using a phospho-specific antibody (Fig 6C). The result was the same for both tumor specimens: signal decreased until disappearing within 24 hours post resection. When an anti-FAK antibody was used to representatively testing protein integrity during time course, it was found that the signal intensity disappeared at 24 hours of incubation (Fig 6C). Still considering Western blotting a non-quantitative assay it is clear that at 24 hours time point protein degradation was maximal.

## Conclusion

The great opportunities offered by the development of the microarray technology have fueled many studies in cancer and more are underway exploiting proteomic tools. Unfortunately, when taking into consideration the reliability of results many factors are usually considered, eg. sample size, treatment homogeneity, type of follow up, while pre-analytical conditions are often neglected. In this study we demonstrate that the likelihood to detect transcriptional differences as well as protein patterns reflecting the biology of samples may be affected by a correct tissue handling.

RNA integrity is not a sufficient proof of a good quality sample and even in samples with high RIN gene expression may be affected by delays in freezing and hypoxia. The stress caused by resection of blood vessels leads in fact to rapid cellular changes typically activating hypoxia or apoptotic pathways. Genes included in signatures commonly used in breast cancer also may undergo transcriptional modulations linked to sample handling procedures. If this is the case misleading results may be obtained for single samples and probably evaluation of the performance of a single gene signature may also be influenced. Interestingly a down-regulation was consistently reported for ESR1, a gene playing a major role in clinical management of breast cancer. The latter finding can probably explain why when looking at messenger levels, the so-called ER gene cassettes are more predictive compared to ER itself [35]. Tissue handling induced modulations are not only confined to messengers. In fact in the case of ESR1 itself also protein expression may be affected by prolonged hypoxia, although both down-regulations [36-38] and up-regulations have been observed [39].

Few studies exist on the stability of proteins and phosphoproteins in tissue after resection and in dependence of preanalytical handling. In this study it was taken into account the dynamic nature of cell protein composition and potentially of cell signaling in post excision setting. Generated data are limited to few specimens and further future in-depth investigation of the fluctuations of protein signaling and post translational modifications is needed. Notwithstanding some phosphoprotein profile fluctuations may potentially be due to tissue individuality, our non-quantitative data are sufficient to support the conclusion that orthovadate addition in lysis buffer is insufficient to block fluctuations in phosphoprotein profiles and also the recommendation to freeze tissue within few minutes of resection to preserve the state of phosphoproteins. In fact, the case of ERBB2 showed the a time-related phosphorylated status in the absence of significant changes at messenger level.

In summary all these data demonstrate that taking advantage of the new profiling techniques for designing tailored treatment strategies is a goal which cannot be achieved without an accurate standardization of tissue collection procedures. Tissue collection procedures should therefore be rigorously defined and controlled to avoid false results which may negatively affect the clinical outcome when attempting personalized treatment strategies. The simple obtainment of good quality RNA samples is not a guarantee for a reliable gene expression profile.

## Abbreviations

IPA: Ingenuity Pathway Analysis; ER: estrogen receptor; RIN: RNA integrity number; qPCR: quantitative PCR.

## Competing interests

The authors declare that they have no competing interests.

## Authors' contributions

LDC carried out the gene profiling studies and participated in the study design and data analysis, VM and SV processed the samples, VC participated in data analysis and drafted the manuscript, IB performed the protein profile analysis and participated in drafting the manuscript, BV is the pathologist in charge of sampling the tumor specimens and confirming the diagnosis, MC performed the statistical and bioinformatics analyses, MAP participated in study design revised the final version of the manuscript and MGD conceived and coordinated the study and approved the manuscript. All Authors read and approved the final manuscript.

## Pre-publication history

The pre-publication history for this paper can be accessed here:

http://www.biomedcentral.com/1471-2407/9/409/prepub
